# Suppression Substractive Hybridization and NGS Reveal Differential Transcriptome Expression Profiles in Wayfaring Tree (*Viburnum lantana* L.) Treated with Ozone

**DOI:** 10.3389/fpls.2016.00713

**Published:** 2016-06-01

**Authors:** Elena Gottardini, Antonella Cristofori, Elisa Pellegrini, Nicola La Porta, Cristina Nali, Paolo Baldi, Gaurav Sablok

**Affiliations:** ^1^Fondazione Edmund Mach, Sustainable Agro-Ecosystems and Bioresources Department, Research and Innovation CentreTrento, Italy; ^2^Department of Agriculture, Food and Environment, University of PisaPisa, Italy; ^3^MOUNTFOR Project Centre, European Forest InstituteTrento, Italy; ^4^Consiglio Nazionale delle Ricerche, Istituto per la Valorizzazione del Legno e delle Specie ArboreeFlorence, Italy; ^5^Fondazione Edmund Mach, Genomics and Biology of Fruit Crops Department, Research and Innovation CentreTrento, Italy; ^6^Plant Functional Biology and Climate Change Cluster (C3), University of Technology SydneySydney, NSW, Australia

**Keywords:** SSH, gene ontology, photosynthesis, detoxification, HSP20-like chaperone, PCR-select™, lipoxygenase activity

## Abstract

Tropospheric ozone (O_3_) is a global air pollutant that causes high economic damages by decreasing plant productivity. It enters the leaves through the stomata, generates reactive oxygen species, which subsequent decrease in photosynthesis, plant growth, and biomass accumulation. In order to identify genes that are important for conferring O_3_ tolerance or sensitivity to plants, a suppression subtractive hybridization analysis was performed on the very sensitive woody shrub, *Viburnum lantana*, exposed to chronic O_3_ treatment (60 ppb, 5 h d^−1^ for 45 consecutive days). Transcript profiling and relative expression assessment were carried out in asymptomatic leaves, after 15 days of O_3_ exposure. At the end of the experiment symptoms were observed on all treated leaves and plants, with an injured leaf area per plant accounting for 16.7% of the total surface. Cloned genes were sequenced by 454-pyrosequencing and transcript profiling and relative expression assessment were carried out on sequenced reads. A total of 38,800 and 12,495 high quality reads obtained in control and O_3_-treated libraries, respectively (average length of 319 ± 156.7 and 255 ± 107.4 bp). The Ensembl transcriptome yielded a total of 1241 unigenes with a total sequence length of 389,126 bp and an average length size of 389 bp (guanine-cytosine content = 49.9%). mRNA abundance was measured by reads per kilobase per million and 41 and 37 ensembl unigenes showed up- and down-regulation respectively. Unigenes functionally associated to photosynthesis and carbon utilization were repressed, demonstrating the deleterious effect of O_3_ exposure. Unigenes functionally associated to heat-shock proteins and glutathione were concurrently induced, suggesting the role of thylakoid-localized proteins and antioxidant-detoxification pathways as an effective strategy for responding to O_3_. Gene Ontology analysis documented a differential expression of co-regulated transcripts for several functional categories, including specific transcription factors (MYB and WRKY). This study demonstrates that a complex sequence of events takes place in the cells at intracellular and membrane level following O_3_ exposure and elucidates the effects of this oxidative stress on the transcriptional machinery of the non-model plant species *V. lantana*, with the final aim to provide the molecular supportive knowledge for the use of this plant as O_3_-bioindicator.

## Introduction

Ozone (O_3_) is a gas naturally present in both troposphere and stratosphere. Particularly, tropospheric O_3_ results from a series of complex photochemical reactions involving primary pollutants, such as nitrogen oxides (NO_*x*_), volatile organic compounds (VOC) and carbon monoxide (CO) mainly generated by human activities (Jenkin and Clemitshaw, 2000). O_3_-producing photochemical reactions are favored by high temperatures and elevated light intensities (Cristofanelli and Bonasoni, 2009). During summertime, the Mediterranean basin is characterized by specific meteorological conditions (i.e., sunny, hot, and dry climate) that enhance photochemical O_3_ formation (Millàn et al., 2000). At the mid-latitudes of the Northern Hemisphere, O_3_ concentrations have more than doubled over the last decades (Monks et al., 2015). Especially in Europe, a general trend toward a decline in peak concentrations has been observed, taking into account the implementation of European air pollution policies reducing precursor emissions. On the other hand, Dawnay and Mills (2009) documented a concomitant increase in O_3_ background concentrations, due to the rise in anthropogenic emissions on a global scale. The ambient O_3_ concentrations have a marked impact not only on human health (Yang and Omaye, 2009; Norval et al., 2011), but also on natural ecosystems, crop productivity (yield and quality), manufactures and works of art (Cass et al., 1989). For these reasons, it is important to understand the regulatory behavior of O_3_ induced stress in plants (e.g., Braun et al., 2014; Doring et al., 2014a; González-Fernández et al., 2014).

After entering into the leaf via the open stomata, O_3_ interacts immediately with biological molecules (like bio-membranes and enzymes) and releases reactive oxygen species (ROS), thereby triggering an oxidative burst (Jaspers et al., 2005). Plants deploy several response mechanisms, some of which are universally conserved among species (Whaley et al., 2015). Through a signaling cascade (Vainonen and Kangasjärvi, 2015), O_3_ affects primarily biological processes involved in plant productivity, such as regulation of structural and chemical components of photosynthesis (Pellegrini et al., 2015), respiration and transpiration (Heath, 2008). The toxicology of this pollutant is complex: many factors such as species, provenance, genotype and leaf age together with environmental, nutritional, and health conditions, play a key role in determining the overall plant response (Manninen et al., 2009). Moreover, contrasting results may be caused by different O_3_ concentrations as well as by different spatial and temporal scales of this pollutant. In this respect, it is important to categorize acute vs. chronic exposures, respectively in short- and long-time treatments (Miller, 2011).

Taking into account the literature, a bio-molecular approach might enable a better understanding of oxidative stress-plant interactions. Current knowledge concerning specific molecular alterations caused by O_3_ at the transcriptomic level is limited (Heath, 2008; Kanter et al., 2013) and has been primarily elucidated (i) in model plants, like *Arabidopsis thaliana* (Hirayama and Shinozaki, 2010) and *Medicago truncatula* (Puckette et al., 2009, 2012); (ii) in crops, like *Pisum sativum* (Savenstrand et al., 2000), *Oryza sativa* (Frei, 2015; Sarkar et al., 2015); and (iii) in herbaceous annual plants, like *Centaurea jacea* (Francini et al., 2008), *Melissa officinalis* (Doring et al., 2014b). Moreover, interactions occurring between O_3_ exposure and changes in the expression profile of several genes have been described in some woody species.

Olbrich et al. (2010) studied transcriptional responses in juvenile *Fagus sylvatica* saplings fumigated with a double concentration of ambient O_3_ over 3 years by conducting microarray hybridization. Recently, the effects of O_3_ (twice ambient concentration) on *F. sylvatica* were investigated performing a large-scale protein analysis based on 2-D Fluorescence Difference Gel Electrophoresis (2-DE DIGE) (Kerner et al., 2014). Rizzo et al. (2007) identified the differential expression of genes induced by an episodic O_3_ treatment (150 ppb for 5 h) in two poplar clones with different O_3_-sensitivity, performing Suppression Subtractive Hybridization (SSH). Nathaniel et al. (2011) investigated the transcriptional and genetics O_3_ responsiveness (chronic and acute treatment) in two divergent *Populus* species. Using an inbred F2 mapping population derived from these two species, they mapped quantitative trait loci (QTLs) associated with O_3_ response, and examined segregation of the transcriptional response to O_3_ and co-localized genes showing divergent responses between tolerant and sensitive genotypes. Tuomainen et al. (1996) studied the acute O_3_-induced reactions at biochemical and transcriptomic levels in two *Betula* clones differing in O_3_-sensitivity, whereas Zinser et al. (2000) focalized on *Pinus sylvestris*. Similarly, Kontunen-Soppela et al. (2010) revealed the patterns of gene expression in *Betula papyrifera* leaves exposed to twice ambient O_3_ concentration using microarray analyses.

In this study, we focused on wayfaring tree (*Viburnum lantana* L.), a common deciduous shrub species, widespread in most part of Europe, North Africa, North America and temperate Asia, and well-known for its sensitivity to O_3_ (Novak et al., 2003; Calatayud et al., 2010). Wayfaring tree sensitivity has been already assessed in terms of morphological (i.e. foliar symptoms) and physiolgical traits in order to evaluate its potential as bio-indicator (Gottardini et al., 2010, 2014a,b). Although a series of studies has been conducted on *Viburnum* spp. (Clement and Donoghue, 2012), this report represents the first attempt to assess the O_3_ sensitivity of this species at molecular level. Specifically, paired SSH and 454-pyrosequencing analyses were performed in plants exposed to near-ambient O_3_ concentrations in controlled environmental conditions. Data collected in this study may be useful to better understand results obtained in natural field conditions.

## Materials and methods

### Cultural practices, plant material, and ozone exposure

One-year-old agamically reproduced saplings of *V. lantana* were grown for 1 month in plastic pots containing a mix of steam sterilized medium soil and peat (1:1) in a controlled environment facility (steady temperature of 20 ± 1°C, relative humidity (RH) of 85 ± 5% and photon flux density at plant height of 530 μmol photon m^−2^ s^−1^ provided by incandescent lamps, following a 14 h photoperiod). A sub-sample of 18 uniform plants were selected when they were ca. 35 cm tall (ca. 30 fully expanded leaves), and were placed in a controlled fumigation environment facility under the same climatic conditions as the growth chamber. Nine plants were exposed to 60 ± 13 ppb of O_3_ (1 ppb = 1.96 μg m^−3^, at 20°C and 101.325 kPa) for 45 consecutive days (5 h d^−1^, in form of a square wave between 9:00 a.m. and 2:00 p.m.). At the same time, nine control plants were exposed to charcoal-filtered air. The entire methodology was performed according to Lorenzini et al. (1994). Leaf samples (n = 5) were collected from three treated and three control plants after 15 days of fumigation (before the onset of foliar symptoms), immediately frozen in liquid nitrogen (N_2_) and kept at −80°C for RNA extraction.

### Symptom assessment

Each marked leaf was scored in percent of O_3_-damaged surface (5%-classes) by in-hand examination with a 10x hand lens and symptoms identified as reported by Gottardini et al. (2014a). For each plant, the mean percentage of injured leaf area of the marked leaves per date was calculated.

### RNA extraction and PCR-select for library creation

Total RNA was extracted from leaves using the protocol described by Gambino et al. (2008). Frozen leaves were ground to fine powder using a pre-chilled mortar and a pestle. Five milliliter of extraction buffer [2% CTAB, 2.5% PVP-40, 2 M NaCl, 100 mM Tris-HCl (pH 8.0), 25 mM EDTA (pH 8.0), and 2% β-mercaptoethanol, added just before use] were heated at 65°C and added to 1 g of ground tissue. After 10 min of incubation at 65°C, two independent extractions were performed, using chloroform:isoamyl alcohol (24:1 v/v). The supernatant was transferred to a new tube and LiCl (3 M final concentration) was added. After 30 min on ice, the RNA was selectively pelleted by centrifugation (21,000 × g for 20 min at 4°C) and re-suspended in 500 μl of SSTE buffer [10 mM Tris-HCl (pH 8.0), 1 mM EDTA (pH 8), 1% SDS, 1 M NaCl], pre-heated at 65°C. An equal volume of chloroform:isoamyl alcohol (24:1 v/v) was added, centrifuged (11,000 × g for 10 min at 4°C) and the supernatant was transferred to a new tube and precipitated with one volume of cold isopropanol. The pellet was washed with ethanol (70%), air-dried and re-suspended in DEPC-treated water. Messenger RNA was isolated using GenElute™ mRNA Miniprep kit (Sigma-Aldrich, St. Louis, MO, USA) according to the manufacturer's instructions. SSH was performed using PCR-select™cDNA subtraction kit (Clontech Laboratories, Mountain View, CA, USA) following the procedure described in the user manual. Forward and reverse subtractions were performed using control and O_3_-treated leaves and PCR products were subsequently sequenced using 454-pyrosequencing (GS FLX+ System, Roche Diagnostics GmbH, Penzberg, Germany). Raw reads obtained from the present study are recorded in the European Nucleotide Archive (ENA) under the project number PRJEB9317.

### Sequence cleaning, processing, and functional annotation

Sequencing reads, obtained from the two libraries (induced and repressed), were cleaned, removing adapter and primer sequences and trimming low-quality ends. We further removed reads shorter than 100 bp and having an average Phred equivalent quality score lower than 15 bp. After read cleaning, homo-polymer stretches (polyA/T) were estimated and were subsequently masked using an in-house PERL script. Masking of the polyA/T was done to increase the sensitivity and specificity in the assembly. For the creation of the Ensembl transcriptome, all the cleaned reads of the induced and repressed libraries were concatenated and a single Ensembl transcriptome was built using MIRA (Chevreux et al., 2004) and CAP3, an overlap layout consensus assembly approach (Huang and Madan, 1999) with an overlap percentage identity cut-off of 97%, to avoid the formation of the spurious assemblies. Resulting contigs and singletons were clustered into representative set of unigenes for each library. All the repetitive reads that were falling into the mega-hub during the MIRA assembly were discarded so as to ensure the correctness and the accuracy of the assembled unigenes.

Following unigene assembly, unigenes in each library were subjected to BLASTx with an *E*-value cut off of 1E-5 against NCBI database available from http://www.ncbi.nlm.nih.gov. All the unigenes were translated into six possible translational frames using the sixpack package of the EMBOSS available from http://emboss.sourceforge.net/ and the putative open-reading frames (ORF) were extracted using the Getorf of the EMBOSS package. All the translated frames were queried for the identification of the InterPro domains and the longest frame with no internal stop codon and with assigned InterPro domain was kept as an assigned functional domain to that respective unigene. FastAnnotator (Chen T. W. et al., 2012) and PLAZA version 2.5 (Van Bel et al., 2013) were run to identify functional annotations associated with the unigenes. Gene Ontology (GO) was derived for each unigenes, slimmed using the plant GO slim (available from http://www.geneontology.org) and classified according to biological and molecular functions and cellular localization. Transcription factors were identified using the PlantTFcat (Dai et al., 2013).

### Expression assessment using read mapping back to assembled ensemble transcriptome

To evaluate the expression levels of the unigenes in induced/repressed libraries, we mapped the individual library reads back to the ensembl transcriptome and reads per kilobase per million (RPKM) was calculated as expression estimate: RPKM (A) = 1,000,000 × C × 1000)/(N × L), where A is defined as the expression of the unigene, C corresponds to the reads that align uniquely to the unigene, N refers to total number of reads that uniquely aligned to all genes, and L refers to the length of gene A. Transcript mapping was performed using BWA-SW algorithm, as implemented in Burrows-Wheeler Aligner [BWA, available from http://bio-bwa.sourceforge.net/; Li and Durbin (2009)]. Expression values were further analyzed to identify the transcripts whose expression was significantly up- or down- regulated during the O_3_ treatment, using the log_2_ fold change (RPKM induced/RPKM repressed), as previously described in Kanter et al. (2013). To identify the functionally and statistically enriched biological pathways and GOs in the up- and down-regulated unigenes, those showing up- and down-regulation in Ensembl unigenes were analyzed using KOBAS, with *A. thaliana* as a background dataset. All the identified biological pathways and GOs were statistically evaluated using the hypergeometric test/Fisher's exact test followed by Benjamini and Hochberg FDR correction [*P* < 0.001 (Mao et al., 2005; Wu et al., 2006; Xie et al., 2011)].

## Results and discussion

### Visible foliar symptoms

After 15 days of O_3_ fumigation leaves did not show any visible symptoms. Thirty days from the beginning of the exposure [AOT40 = 3000 ppb h; AOT40: ozone Accumulated Over a Threshold of 40 ppb, *sensu* de Leeuw and van Zantwoort (1997)], fully expanded leaves from O_3_-fumigated plants showed several minute (Ø 1-2 mm) roundish dark-blackish necrosis located in the interveinal area of the adaxial surface. Symptoms were observed on all the examined leaves (*n* = 30) and plants (*n* = 6), with an injured leaf area per plant accounting for 4.2% (SE 1.4%; range 1–20%). At the end of the experiment (AOT40 4500 ppb h, 45 days from the beginning of exposure), the injured area was 16.7% (SE 2.47%; range 5–60%) of the total surface. Visible foliar injury has been used in many field experiments as an indicator of the response of *V. lantana* to O_3_ exposure (e.g., Gottardini et al., 2010). Fully expanded leaves showed symptoms similar to those previously reported in seedlings from OTC experiments (Novak et al., 2005, 2008) and in plants grown in natural conditions (Gottardini et al., 2014a,b).

### Read generation and *De novo* assembly for plants exposed to O_3_ stress

A total of 43,815 and 13,610 high quality reads were generated in induced and repressed libraries, respectively (Table 1). Following sequencing, reads were filtered as described in the Materials and Methods section, resulting in a total of 38,800 and 12,495 clean reads (Table 1). The mean read length for the induced and repressed library was 319 ± 156.7 and 255 ± 107.4 bp, respectively (*data not shown*). 454-assembly using MIRA of the Ensembl transcriptome yielded a total of 1238 unigenes with a total sequence length of 389,126 bp and an average length size of 389 bp (GC = 49.9%, Table 1). ORF predictions revealed a total of 161 (13.0%) sequences with a proper start codon and a total of 574 sequences (46.3%) with proper stop codons. Interestingly, a low number of unigenes (73, 5.9%) with predicted ORF contain frameshifts, which indicates a good quality of the assembly. Identified frameshifts were corrected using FrameDP (Gouzy et al., 2009).

**Table 1:** **Summary of RNA sequencing and *de novo* assembly to construct the gene set of *Viburnum lantana* plants exposed to O_3_ treatment (60 ppb of O_3_, 5 h d^−1^ for 15 consecutive days)**.

	**Induced**	**Repressed**	**Ensembl**
Initial sequencing reads	43815	13610	57425
Cleaned reads	38800	12495	51295
Unigenes	543	705	1238
Total length of Unigenes (bp)	137438	254585	389126
N50 stats (pb):	277	434	389
Total GC count (pb):	68866	126700	194216
GC (%):	50.1	49.8	49.9
GO categories	262 (48.3%)	481 (68.2%)	744 (60.1%)
Functional protein domains	295 (54.3%)	476 (67.5%)	772 (62.4%)

Number of the sequencing reads, clustering details, and the unigene details. GC, guanine-cytosine content; GO, gene ontology.

### Functional classification for plants exposed to O_3_

**Figure 1:**
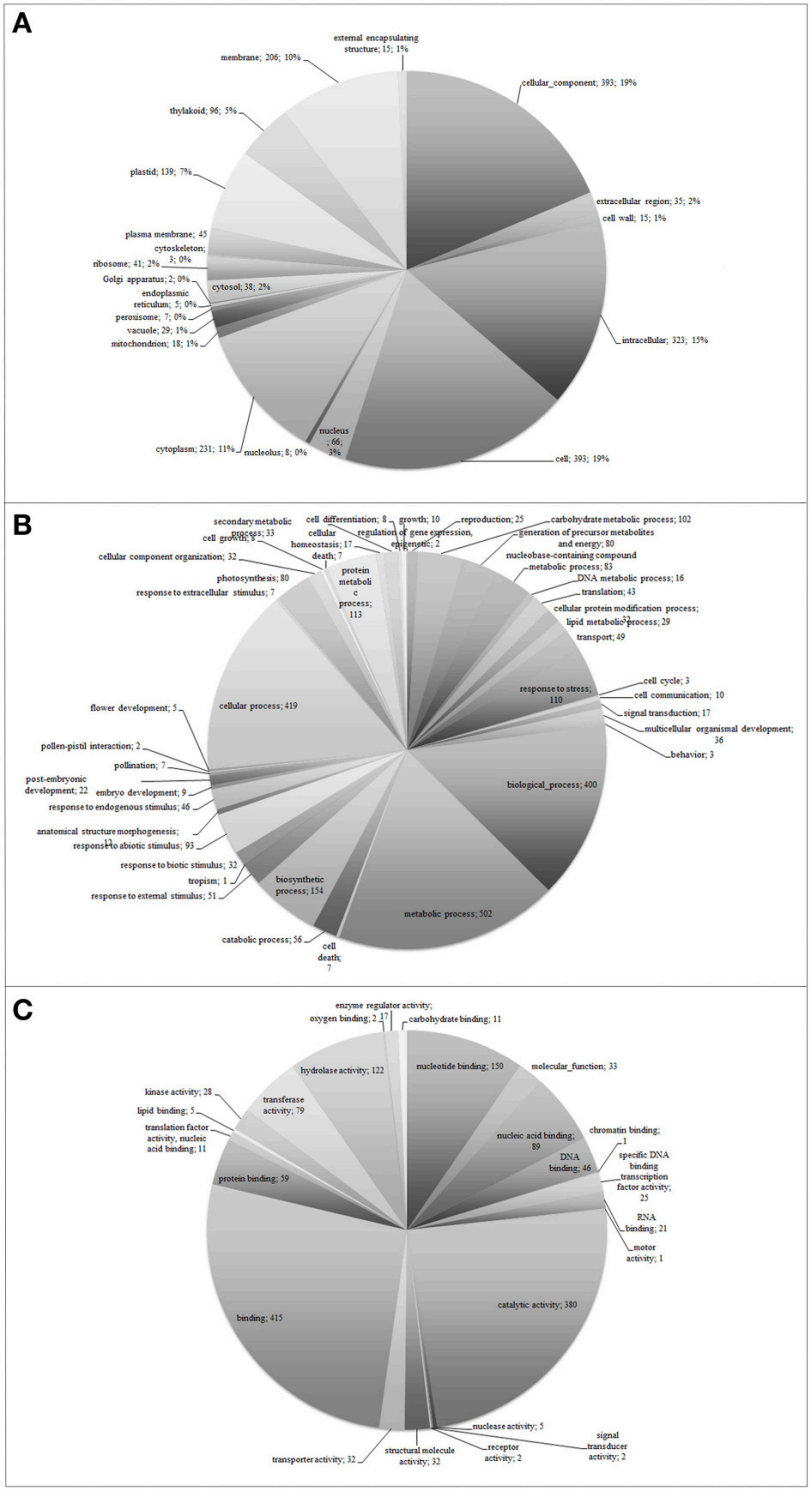
**Functional classification of *Viburnum lantana* ensembl unigenes within cellular component (A), biological process (B), and molecular function (C) categories**.

Functional classification of the assembled unigenes indicates the putative functional changes occurring at gene level in plants subjected to O_3_. The Ensembl transcriptome was annotated by performing stringent BLASTx searches (*E*-value threshold, 1E-5) against NCBI and PLAZA version 2.5. Functional annotation of the Ensembl transcriptome (1,238 unigenes) revealed a total of 744 transcript sequences (60.1%), with an assigned GO category and a total of 772 transcript sequences (62.4%), with an assigned InterPro domain, respectively (Table 1 and Supplementary Table 1 and Supplementary Datasheet 1). The results of BLASTx (*E*-value threshold, 1E-5) searches against NCBI database and PLAZA version 2.5 (Van Bel et al., 2013) resembled those reported for whole genome expressed genes and tissue-specific cDNA extracts in other plant species (Legrand et al., 2007, 2010; Hao et al., 2015). Based on GO-slim annotations, Ensembl unigenes were classified into three ontological categories: cellular component, biological process, and molecular function (Figure 1). Within the cellular component category, 21 GO slims were identified including cell, membrane, thylakoid, and other apparatus (Figure 1A). Cell, cellular component, intracellular, and cytoplasm were the most represented slims in terms of number of genes (393, 393, 323, and 231, respectively). On the other hand, 42 GO slims were recognized within the biological process category, such as translation, reproduction, transport, photosynthesis, cell death, and other functions (Figure 1B). Among all these slims, metabolic and cellular processes were the most frequent ones (502 and 419 genes, respectively). Aside from GO cellular processes, the response to stress was the next most abundant GO slim (110 gene numbers) after biological and biosynthetic process (400 and 154, respectively). It comprised responses to different stresses, including abiotic and endogenous stimuli (93 and 46 genes, respectively, Figure 1B). Within the molecular function category, 24 GO slims were observed including protein binding, transcription regulator activity, carbohydrate binding, hydrolase activity, receptor activity, and other enzymatic function (Figure 1C). Among all of these slims, binding and catalytic activity appeared more frequently (415 and 380, respectively).

### Expression estimates and functional profiling of up- and down-regulated unigenes

Expression abundance for the unigenes was estimated by mapping the reads from the individual library to the assembled Ensemble transcriptome and was assessed as number of reads per RPKM. According to Kanter et al. (2013), prior to linking the expression estimates to biological functions, unigenes having a RPKM lower than 7 were discarded to avoid the false interpretation of the log_2_ fold change values. A total of 78 (6.3%) Ensembl unigenes showed a variation in expression values after O_3_-treatment. In particular, 41 ensembl unigenes showed an up-regulation average [log_2_ (RPKM induced/RPKM repressed) of 3.7 (2.1 SE) ranged between 0.4 and 8.7 (Table 2)] and the other 37 showed a down-regulation [log_2_ (RPKM induced/RPKM repressed) ranged between –0.1 and –8.7 (Table 3)]. Functional ontology and the assigned InterPro domain of the up- and down-regulated unigenes (30 and 29, respectively) are tabulated in the Supplementary Table 2. We observed induction of unigenes (EnsVib0416 and EnsVib0041), which are functionally associated to heat-shock proteins (IPR008978, HSP20-like chaperone; IPR002068, Heat shock protein Hsp20; Supplementary Table 2). The expression levels of HSPs (IPR008978 and IPR002068) in terms of RPKM for the induced library were 58,328.8 and 12,510.56 (vs. 140.4 and 212.3, for the repressed library respectively), which clearly indicate a strong induction of HSPs in O_3_-treated plants [log_2_ fold 8.7 and 5.9, respectively (Table 2)]. The observed results are in accordance with previous observations reported by Eckey-Kaltenbach et al. (1997) in *Petroselinum crispum* seedlings exposed to O_3_ (200 ppb, 10 h) and post-cultivated in pollutant-free air, using a Northern blot analysis. Recently, Al-Whaibi (2011) reviewed the role of HSPs demonstrating their function as molecular chaperones, regulating the (i) localization, (ii) degradation, (iii) accumulation, and (iv) folding of proteins during their synthesis. Taking into account these studies, HSPs can be considered as the first line of defense against O_3_ (Haslbeck and Vierling, 2015).

**Table 2:** **Expression log_2_(RPKM induced/RPKM repressed) of the up-regulated Ensembl unigenes having homologous gene families according to PLAZA version 2.5 and associated Interpro domains**.

**Ensembl Unigene**	**Homologous Gene Families**	**Associated InterPro domains**	**Log_2_ (RPKM induced/RPKM repressed**
EnsVib0002	HOM000181	IPR001024 IPR008976 IPR001246 IPR000907 IPR013819	7.1
EnsVib0022	HOM000082	IPR009038 IPR000348	2.4
EnsVib0041	HOM000926	IPR008978 IPR002068	5.9
EnsVib0046	HOM005074	IPR008892	1.0
EnsVib0120	–	–	0.7
EnsVib0128	HOM000463	IPR002912	3.6
EnsVib0140	HOM000058	IPR013128 IPR000668 IPR013201	2.2
EnsVib0155	HOM000058	IPR013128 IPR000668 IPR013201	1.4
EnsVib0159	HOM002664	IPR002020 IPR016142 IPR016141	5.5
EnsVib0185	HOM001028	IPR017936 IPR012335 IPR000866 IPR012336	0.4
EnsVib0225	–	–	2.5
EnsVib0241	HOM000826	IPR012677 IPR000504	5.1
EnsVib0243	HOM000826	IPR012677 IPR000504	6.5
EnsVib0251	–	–	5.6
EnsVib0267	HOM002675	IPR000772 IPR001574 IPR017989 IPR016139 IPR016138 IPR008997	5.3
EnsVib0271	–	–	1.2
EnsVib0273	HOM000085	IPR012269 IPR000425	4.0
EnsVib0283	–	–	2.5
EnsVib0308	–	–	4.5
EnsVib0314	HOM003594	IPR004662 IPR011148 IPR001057 IPR001048	3.6
EnsVib0316	–	–	1.0
EnsVib0332	–	–	4.3
EnsVib0341	HOM000031	IPR000109 IPR016196	5.0
EnsVib0359	–	–	4.7
EnsVib0376	HOM000016	IPR002213	5.9
EnsVib0383	HOM000438	IPR007608	4.5
EnsVib0387	HOM000533	IPR008580	4.7
EnsVib0402	–	–	5.9
EnsVib0416	HOM000926	IPR008978 IPR002068	8.7
EnsVib0419	HOM000451	IPR005016	4.9
EnsVib0421	HOM002452	IPR003823	1.4
EnsVib0469	HOM000198	IPR012335 IPR002109 IPR012336 IPR014756 IPR003172	1.0
EnsVib0492	HOM004549	IPR011990 IPR006597	3.4
EnsVib0505	HOM000025	IPR003959 IPR005936 IPR003593 IPR000642	5.1
EnsVib0516	HOM000393	IPR012335 IPR010987 IPR004046 IPR012336 IPR004045 IPR017933	5.3
EnsVib0535	HOM001845	IPR008962 IPR002110 IPR000535 IPR020683	0.5
EnsVib0765	HOM005953	IPR014946	3.5
EnsVib0767	–	–	3.3
EnsVib0812	HOM000507	IPR016040 IPR001891 IPR012301 IPR012302	1.5
EnsVib0830	HOM000080	IPR014778 IPR012287 IPR017930 IPR006447 IPR009057	4.9
EnsVib0869	HOM000033	IPR013525 IPR017871 IPR003593 IPR003439	1.5

RPKM, kilo base of exon model per million mapped reads.

**Table 3:** **Expression log_2_(RPKM induced/RPKM repressed) of the down-regulated Ensembl unigenes having homologous gene families according to PLAZA version 2.5 and associated Interpro domains**.

**Ensembl Unigene**	**Homologous Gene Families**	**Associated InterPro domains**	**Log_2_(RPKM induced/RPKM repressed)**
EnsVib0016	HOM000635	IPR020568 IPR000754 IPR014721	−4.6
EnsVib0032	HOM005074	IPR008892	−1.3
EnsVib0035	HOM000181	IPR001024 IPR008976 IPR001246 IPR000907 IPR013819	−2.2
EnsVib0036	HOM000181	IPR001024 IPR008976 IPR001246 IPR000907 IPR013819	−8.7
EnsVib0039	HOM000950	IPR011032 IPR013149 IPR013154 IPR016040 IPR002085 IPR020843	−0.1
EnsVib0071	–	–	−1.6
EnsVib0080	HOM004197	IPR006311 IPR008797	−5.7
EnsVib0083	HOM000339	IPR020478 IPR010979 IPR001892 IPR001965 IPR011011 IPR017956	−3.2
EnsVib0085	HOM001274	IPR003959	−1.3
EnsVib0139	HOM000013	IPR018957 IPR001841	−0.1
EnsVib0148	HOM000250	IPR001395	−0.6
EnsVib0162	HOM000005	IPR001128 IPR017973 IPR002401	−6.0
EnsVib0178	HOM000181	IPR001024 IPR008976 IPR001246 IPR000907 IPR013819	−5.0
EnsVib0199	HOM001274	IPR003959	−5.4
EnsVib0212	HOM004197	IPR006311 IPR008797	−3.8
EnsVib0217	–	–	−0.2
EnsVib0244	HOM000648	IPR013785 IPR000741	−3.6
EnsVib0266	HOM001274	IPR003959	−4.4
EnsVib0284	HOM003875	IPR009346	−1.6
EnsVib0313	HOM000858	IPR000894	−5.3
EnsVib0435	HOM000051	IPR016040 IPR002347 IPR002198	−0.8
EnsVib0445	HOM005189	IPR017498	−6.1
EnsVib0462	HOM000934	IPR013845 IPR000876 IPR013843 IPR005824 IPR002942	−2.2
EnsVib0476	HOM001646	IPR020568 IPR000851 IPR013810 IPR005324 IPR014721 IPR018192	−1.1
EnsVib0499	–	–	−1.4
EnsVib0501	HOM001714	IPR006214	−4.5
EnsVib0509	–	–	−5.1
EnsVib0512	HOM002153	IPR008991 IPR018259 IPR001147	−8.5
EnsVib0518	HOM000034	IPR012334 IPR000070 IPR006501 IPR011050	−7.2
EnsVib0638	HOM002856	IPR006082	−1.2
EnsVib0688	HOM000502	IPR018957 IPR001650 IPR001841 IPR014021 IPR014001 IPR000330	−0.6
EnsVib0735	HOM003079	IPR003095 IPR015609 IPR001623	−2.6
EnsVib0746	HOM000011	IPR005123	−2.6
EnsVib0792	–	–	−2.6
EnsVib0899	–	–	−0.6
EnsVib0984	–	–	−1.3
EnsVib1062	–	–	−1.6

RPKM, kilo base of exon model per million mapped reads.

Using a proteomic approach, Torres et al. (2007) documented that expression levels of HSPs were strongly increased in O_3_-stressed young leaves of maize (200 ppb, 3 h) and that their functions were correlated with glycolysis, photosynthesis, antioxidant-, and pathogen-related defense. It is worthwhile to mention that HSPs (especially HSP20 superfamily) play a pivotal role in protecting the photosynthetic machinery against damage caused by photo-oxidative stress (Lee et al., 2000). Although this property has been demonstrated during heat stress, we can suppose that the induction of HSPs by O_3_ treatment could (i) increase the resistance of photosynthetic machinery to photoinhibition, and (ii) affect stress tolerance.

We observed a log_2_ fold up-regulation (RPKM induced/RPKM repressed = 7.1) of a unigene (EnsVib0002) functionally associated to lipoxygenase activity (GO:0016165, Supplementary Table 2). This is supported by similar findings in *Lens culinaris* seedlings exposed to O_3_ flux after 30 min from the beginning of the treatment (Maccarrone et al., 1997). In soybean seedlings, O_3_ up-regulated the lipoxygenase gene and its activity, with a concomitant enhanced membrane lipid peroxidation (Maccarrone et al., 1992). Recent RNA-seq based transcriptomics indicated an increased expression of genes involved in lipid metabolic process in two soybean varieties exposed to an episodic O_3_ treatment [25–75 ppb, 4 h (Whaley et al., 2015)]. Plant lipoxygenases, which use molecular oxygen to produce hydroperoxides from unsaturated fatty acids, play a key role in (i) growth and development, (ii) senescence, and (iii) responses to biotic and abiotic stresses. In particular, lipoxygenase activity has been implicated in membrane alteration and could mediate the O_3_-effect. It is worthwhile to mention that lipid oxidation is a double-edged event due to (i) its damaging effects on lipids and membrane components and (ii) a putative beneficial role in the signaling pathway [e.g., jasmonic acid formation, Vaultier and Jolivet (2015)].

An induction of unigenes (EnsVib0516) functionally associated with glutathione (GSH) was observed with GO GSH binding (GO:0043295), GSH transferase (GO:0004364), and GSH peroxidase activities (GO:0004602, Supplementary Table 2), according with the earlier reports by Tosti et al. (2006) in *A. thaliana* ecotype Columbia (Col-0) plants exposed to O_3_ (300 ppb, 6 h) after 3 h from the beginning of the treatment. Antioxidant enzymatic activities were expected to rise during a situation that leaded to increased oxidative stress. Results indicate that GSH-dependent detoxification pathways were induced by O_3_. Specifically, the expression level of GSH in terms of RPKM for the induced library was 1,198.5 (vs. 312.6 for the repressed library), which suggests an involvement of disulphide bridges in redox-control process. This is in agreement with the results reported by D'Haese et al. (2006) for *A. thaliana* plants exposed to an episodic O_3_ treatment (150 ppb, 8 h).

A repression of unigenes (EnsVib0080 and EnsVib0212) functionally associated to photosynthetic process with GO: photosystem (GO:0009521) and photosynthesis (GO:0015979, Supplementary Table 2) was found. The expression levels of photosynthetic process in terms of RPKM for the induced library were 95.8 and 368.2 (vs. 4,909.0 and 5,013.0, for the repressed library respectively), clearly suggesting a decrease in photosynthetic performance in O_3_-treated plants [log_2_ (RPKM induced/RPKM repressed) = −5.8 and −3.8, respectively (Table 3)]. Similarly, Kontunen-Soppela et al. (2010) reported a decreased expression of photosynthesis- and carbon fixation-related genes in *B. papyrifera* plants exposed to O_3_ alone (2x ambient O_3_ concentration) or in combination with CO_2_ (target 550 ppm), during the growing season since 1998. A previous microarray analysis indicated that many genes involved in photosynthesis were down-regulated in *Populus tremuloides* plants (clone 216) subjected to chronic O_3_ fumigation (1.5x ambient O_3_ concentration for 5 consecutive years; Gupta et al., 2005).

Oxidation reactions would be expected to reduce net photosynthesis predisposing plants to the inhibition of PSII electron transport (Pellegrini, 2014) and possibly, accelerating the onset of cell senescence (Pellegrini et al., 2015). In the present study, impaired photosynthesis was seen as a down-regulation of PSII oxygen-evolving complex (OEC) PsbQ genes (regulators for the biogenesis of optically active PSII). According to Gururani et al. (2015), the down-regulation of OEC is an efficient and dynamic feedback mechanism to (i) reduce/regulate the generation of reactive oxygen radicals in PSII (favoring electron donation by non-water electron donors with a high rate constant) and (ii) to provide protection against photodamage in response to abiotic stress. In our case, the easy accessibility of non-water electrons from antioxidant molecules and the concomitant increase of GSH-related genes confirm that plants use a specific set of active mechanisms for ROS scavenging during O_3_ exposure.

To identify the functional enriched terms and pathways and to approximate the coverage of sequenced and assembled unigenes, we compared the identified 78 unigenes, whose expression altered during the O_3_ stress in *V. lantana* to *A. thaliana* genome predicted coding sequences (CDS) using KOBAS (*E*-value threshold, 1E-08; Supplementary Figure 1). Interestingly, 45 out of 78 unigenes showed putative functional orthologs in *A. thaliana* (Table 4). GO enrichment was observed according to the observed functionally enriched linoleate 13S-lipoxygenase activity (GO:0016165, *P* = 1.86E-07, corrected *P* = 1.59E-05), which might indicate the conversion of the linoleate into 13-HPODE, responsible for the activation of the lipid peroxidation process. This suggests that O_3_ treatments may induce deleterious effects on (i) integrity, (ii) conformation, and (iii) transport capacity of membranes in *V. lantana*, as reported in other species (Yan et al., 2010; Pellegrini et al., 2011). Dynamics of gas exchange results strongly altered as confirmed by the enrichment of GO categories related to thylakoid (GO:0009579, GO:0009535, GO:0055035, GO:0044436, GO:0009534, GO:0031976). It confirms that O_3_ exposure, by free-radical production, induces alterations in the (i) photosynthetic apparatus, (ii) content/pattern of thylakoid, and (iii) functional state of chloroplast membranes. Particularly, O_3_-derivative molecular species induce changes in gene expression responsible for rearrangements in the thylakoid architecture of *V. lantana* leaves limiting the damage of PSII activity (as a compensatory mechanism for the inhibiting photosynthetic effects) and, generally, counteracting the oxidative stress.

**Table 4:** **Statistical analysis on functional homologs (GO categories) between *Viburnum lantana* and *Arabidopsis thaliana* ontology assignment and enrichment analysis using hypergeometric test/Fisher's exact test and corrected values after Benjamini and Hochberg FDR correction**.

**Functional Term**	**Gene Ontology**	***P*-value**	**Corrected *P*-value**
Photosynthetic membrane	GO:0034357	3.63E-11	2.02E-08
Thylakoid	GO:0009579	3.94E-11	2.02E-08
Chloroplast thylakoid membrane	GO:0009535	1.65E-10	3.37E-08
Plastid thylakoid membrane	GO:0055035	1.73E-10	3.37E-08
Response to desiccation	GO:0009269	1.79E-10	3.37E-08
Thylakoid part	GO:0044436	1.97E-10	3.37E-08
Thylakoid membrane	GO:0042651	3.28E-10	4.80E-08
Chloroplast thylakoid	GO:0009534	9.10E-10	1.04E-07
Plastid thylakoid	GO:0031976	9.10E-10	1.04E-07
Organelle subcompartment	GO:0031984	1.02E-09	1.04E-07
Lipoate metabolic process	GO:0009106	1.62E-07	1.51E-05
Linoleate 13S-lipoxygenase activity	GO:0016165	1.86E-07	1.59E-05
Response to herbivore	GO:0080027	4.34E-07	3.43E-05
Glycine catabolic process	GO:0006546	6.30E-07	4.61E-05
Serine family amino acid catabolic process	GO:0009071	7.82E-07	5.34E-05
Glycine metabolic process	GO:0006544	9.61E-07	6.16E-05
Serine family amino acid metabolic process	GO:0009069	1.42E-06	7.68E-05
Oxidoreduction coenzyme metabolic process	GO:0006733	1.42E-06	7.68E-05
Divalent metal ion transport	GO:0070838	2.16E-06	1.11E-04
Divalent inorganic cation transport	GO:0072511	2.34E-06	1.14E-04
Chloroplast envelope	GO:0009941	2.61E-06	1.21E-04
Unsaturated fatty acid biosynthetic process	GO:0006636	3.40E-06	1.45E-04
Unsaturated fatty acid metabolic process	GO:0033559	3.40E-06	1.45E-04
Plastid envelope	GO:0009526	4.11E-06	1.65E-04
Cellular cation homeostasis	GO:0030003	5.25E-06	2.07E-04
Cellular ion homeostasis	GO:0006873	7.37E-06	2.07E-04
Oxidoreductase activity	GO:0016702	7.40E-06	2.07E-04
Chloroplast stroma	GO:0009570	8.42E-06	2.98E-04
Oxylipin biosynthetic process	GO:0031408	8.85E-06	3.02E-04
Cellular chemical homeostasis	GO:0055082	1.02E-05	3.36E-04
Coenzyme biosynthetic process	GO:0009108	1.07E-05	3.34E-04
Plastid stroma	GO:0009532	1.13E-05	3.46E-04
Vitamin metabolic process	GO:0006766	1.22E-05	3.46E-04
Oxylipin metabolic process	GO:0031407	1.23E-05	3.46E-04
Coenzyme metabolic process	GO:0006732	1.25E-05	3.46E-04
Sulfur amino acid metabolic process	GO:0000096	1.72E-05	4.65E-04
Cellular homeostasis	GO:0019725	1.79E-05	4.70E-04
Envelope	GO:0031975	1.96E-05	4.90E-04
Organelle envelope	GO:0031967	1.96E-05	4.90E-04
Chloroplast part	GO:0044434	2.02E-05	4.93E-04
Response to temperature stimulus	GO:0009266	2.08E-05	4.95E-04
Photosynthesis	GO:0015979	2.23E-05	5.19E-04
Cellular amino acid biosynthetic process	GO:0008652	2.30E-05	5.24E-04
Plastid part	GO:0044435	2.66E-05	5.78E-04
Cation homeostasis	GO:0055080	2.71E-05	5.78E-04

Only GO categories with P < 0.001 are showed (45 on the total 78 found for Viburnum lantana).

Using RNA-seq, Liu et al. (2015) indicated the abundance and enrichment of the differentially expressed genes involved in the thylakoid, plastid part, chloroplast, and plastids envelope in *Reaumuria soongorica* leaves subjected to UV-B radiation. Additionally, previous immunological studies documented that O_3_ treatment could affect energy transfer processes by inducing alterations in thylakoid membrane proteins (Tognini et al., 1997; Ranieri et al., 2000). Following O_3_ treatment, a complex sequence of events takes place in the cells of *V. lantana* (at intracellular and membrane level), altering key biological processes [such as metabolism, protein fate (folding, modification, and destination) and transports] and molecular functions (catalytic and hydrolase activities). This suggests that plants react to O_3_ changing metabolic processes (for example lipid catabolism, sugar and amino acid metabolism) that are both used (i) directly, as alternative sources of energy, (nitrogen and carbon skeletons) and (ii) indirectly, as substrates of secondary metabolite modifications. According to Heath (2008), we can conclude that O_3_ induces deep changes in the expression of genes responsible for biochemical adjustments and metabolic shifts.

### Transcription factors

Thirty-four families of transcription factors were observed for Ensembl unigenes (Supplementary Table 3). Myeloblast (MYB)-factors were previously described to be associated with a diverse array of cellular responses, including plant secondary metabolism as well as biotic and abiotic tolerance (Kwon et al., 2013) and were observed in the induced library. MYB proteins responded at the transcriptional level to O_3_ stress in *V. lantana* (EnsVib0607_ORF+3, IPR001005, and IPR009057 domains). MYB transcription factors could have repressing effects on genes involved in the biosynthesis of phenylpropanoids (Bender and Fink, 1998), flavonoids (Borevitz et al., 2000), auxin and consequently have an effect on the maintenance of cell wall development, cuticle formation, and lipid metabolism. Few studies based on the evaluation of plant responses to drought, salt, and UV stress (Hemm et al., 2001; Golldack et al., 2014) have reported the functional role of MYBs.

In our study, WRKY-factors were found, which have been previously reported to be important components in the complex signaling processes during plant stress responses (Dong et al., 2003; Zhang et al., 2015). Far less information is available to understand the function of WRKY proteins in abiotic stress. Some studies demonstrated that the expression of many WRKY genes is greatly and rapidly induced in response to wounding, temperature, nutrient deficiency, drought, and salinity (Chen L. et al., 2012). WRKY proteins respond to O_3_ stress at the transcriptional level in *V. lantana* plants (EnsVib0769_ORF-1, IPR003657 domain) and WRKY transcription factors could have inducing effects on genes involved in O_3_ perception/signal transduction pathway and in redox regulation. This result suggests that WRKY-factors could act as redox-responsive sequences and, consequently, as promoter elements specific for redox regulation (since they possess a redox-sensitive zinc-finger DNA binding domain). Similar findings were reported by Tosti et al. (2006) in Col-0 *Arabidopsis* plants 3 h after the beginning of O_3_ treatment. SSH analysis indicated that WRKY genes may be involved in redox regulation in two poplar hybrid clones exposed to an episodic O_3_ treatment (Rizzo et al., 2007). Similarly, Mahalingam et al. (2003) documented an over-representation of WRKY motifs in the promoter region of genes up-regulated by an episodic O_3_ exposure (350 ppb, 6 h) in Col-0 *Arabidopsis* plants. Xu et al. (2015) observed that four WRKY-transcriptional factors genes were highly induced by O_3_ treatment (350 ppb, 2 h) in Col-0 *Arabidopsis* plants. Furthermore, the expression gene profile after O_3_ fumigation was similar to that of tomato after *Botrytis cinerea* (a fungus) (Journot-Catalino et al., 2006) and *Pseudomonas syringae* (a bacterium) infections (Birkenbihl et al., 2012), suggesting that (i) O_3_ resembles a biotic elicitor and (ii) stress-regulated genes represent a general stress response.

## Conclusions

This is the first study on differentially expressed genes after O_3_ treatment in *V. lantana* plants. A large number of genes involved in signaling/transcription, stress/defense, and protein metabolism showed significant differences in expression of plants exposed to chronic O_3_ treatment, suggesting that complex molecular alterations occurred. By GO slims and pathways enrichment of the co-regulated genes, it could be demonstrated that following O_3_ exposure, a complex sequence of events takes place in the cells at intracellular and membrane level, altering a series of biological processes [such as metabolism, protein fate (folding, post-translational modification, and destination) and transport] and molecular functions (catalytic and hydrolase activities). Specifically, the down-regulation of genes associated to photosynthesis demonstrates the deleterious effects of O_3_. Up-regulation of genes involved in antioxidant-detoxification pathway and thylakoid-localized proteins may be an effective strategy of defense against O_3_. This research can be considered as an useful basis to (i) generate the functional resources for the putative characterization of identified unigenes in *V. lantana* and (ii) better understand the response to O_3_ exposure in a non-model species.

## Author contributions

The work presented here was carried out in collaboration among all authors. NL and CN defined the research theme and obtained funding. EG, AC, EP, PB, and GS designed methods, carried out laboratory experiments, and analyzed the data. AC, EP, and GS co-designed experiments, discussed analyses, interpreted the results, and wrote the paper. All authors have contributed to discuss the results and implications of the work and to comment on the manuscript at all stages before approvation.

### Conflict of interest statement

The authors declare that the research was conducted in the absence of any commercial or financial relationships that could be construed as a potential conflict of interest.

## Supplementary material

Supplementary Figure 1 **Statistical pathway enrichment of the differentially expressed genes against *Arabidopsis thaliana***.

Supplementary Table 1 **Table showing the GO and associated InterPro annotations with ensembl transcripts showing the up- and down-regulations during the ozone treatment**.

Supplementary Table 2 **Detailed classification of expression log_2_(RPKM induced/RPKM repressed) of the up- and down-regulated Ensembl unigenes having homologous gene families according to PLAZA version 2.5 and associated InterPro domains**. Abbreviations: RPKM: kilo base of exon model per million mapped reads. Detailed Classification of the associated functional categories associated with the expressed transcripts in ozone treatment.

Supplementary Table 3 **Table showing the family and associated InterPro domains of ensembl transcription factors**.
