# Supplementary figures and images for: Suppression Substractive Hybridization and NGS Reveal Differential Transcriptome Expression Profiles in Wayfaring Tree (Viburnum lantana L.) Treated with Ozone
Source: Front Plant Sci. 2016 Jun 1;7:713. doi: 10.3389/fpls.2016.00713 (PMC4887494; doi:10.3389/fpls.2016.00713)

## Slide 1
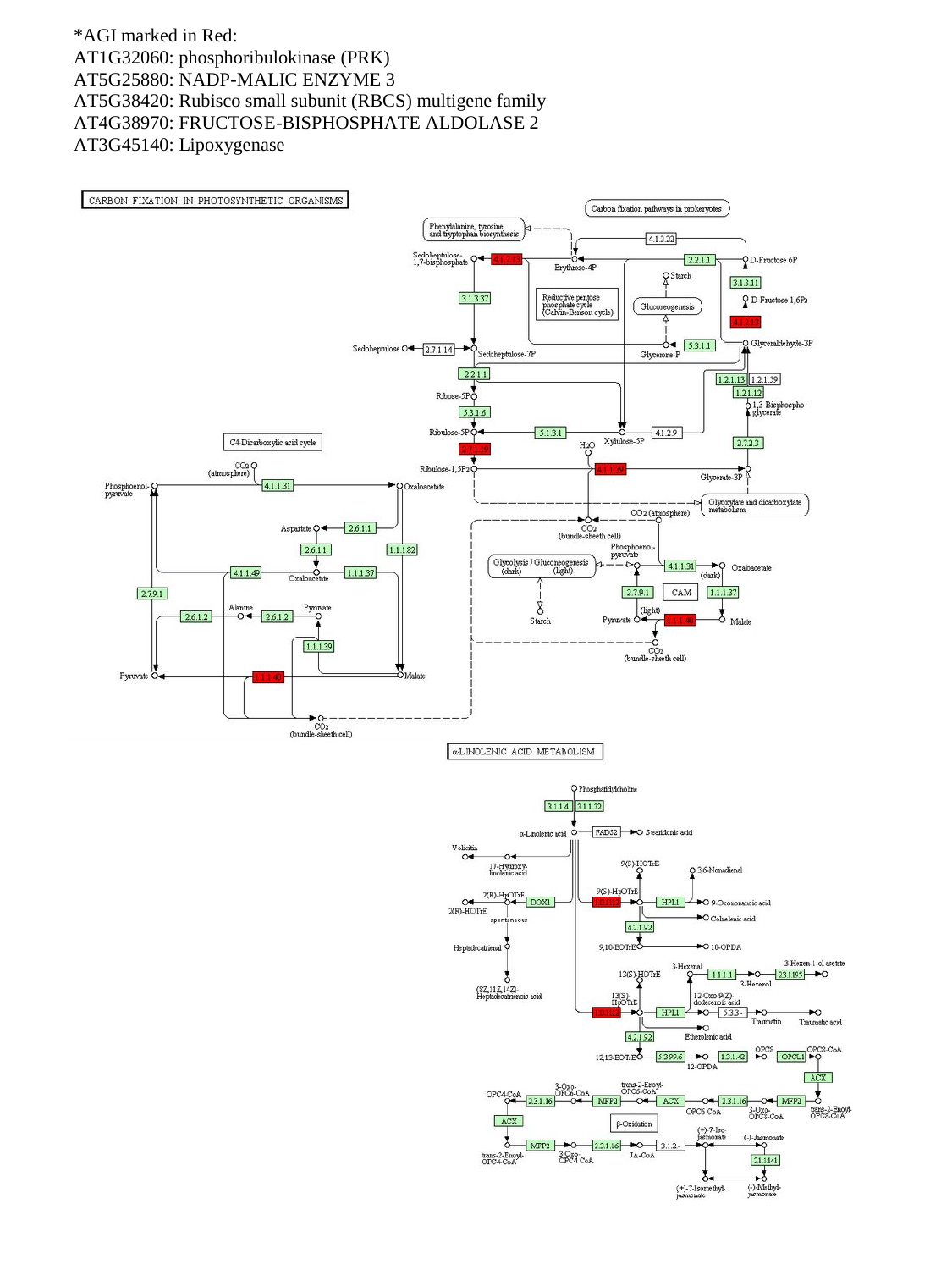

Supplement: Supplementary Figure 1 — Statistical pathway enrichment of the differentially expressed genes against Arabidopsis thaliana. [file Presentation1.PPTX]
